# Supplementary material for: Synergistic effects of climate and landscape change on the conservation of Amazonian lizards
Source: PeerJ. 2022 Mar 29;10:e13028. doi: 10.7717/peerj.13028 (PMC8973465; doi:10.7717/peerj.13028)
Supplement: Supplemental Information 1 — Points: number of records (4.5 × 4.5 km pixel) and AUC and TSS values. [file peerj-10-13028-s001.docx]

**Table S1:**

**Lizard species analyzed.**

Lizards species. Points: number of records (4.5 × 4.5 km pixel) and AUC and TSS values.

| **Species** | **Points** | **AUC** | **TSS** |
| --- | --- | --- | --- |
| **Phyllodactylidae** |  |  |  |
| *Thecadactylus solimoensis* Bergmann & Russell, 2007 | 63 | 0.996 | 0.743 |
| **Sphaerodactylidae** |  |  |  |
| *Chatogeckko amazonicus* (Andersson, 1918) | 223 | 0.993 | 0.767 |
| *Gonatodes annularis* Boulenger, 1887 | 23 | 0.992 | 0.735 |
| *Gonatodes hasemani* Griffin, 1917 | 55 | 0.997 | 0.730 |
| *Gonatodes humeralis* (Guichenot, 1855) | 378 | 0.995 | 0.755 |
| *Lepidoblepharis heyerorum* Vanzolini, 1978 | 44 | 0.997 | 0.866 |
| *Pseudogonatodes guianensis* Parker, 1935 | 51 | 0.995 | 0.817 |
| **Mabuyidae** |  |  |  |
| *Copeoglossum nigropunctatum* (Spix, 1825) | 361 | 0.994 | 0.798 |
| *Varzea altamazonica* (Miralles, Barrio-Amorós, Rivas & Chaparro-Auza, 2006) | 41 | 1 | 0.829 |
| *Varzea bistriata* (Spix, 1825) | 46 | 0.999 | 0.836 |
| **Dactyloidae** |  |  |  |
| *Dactyloa punctata* (Daudin, 1802) | 177 | 0.997 | 0.825 |
| *Dactyloa transversalis* (Duméril in Duméril & Duméril, 1851) | 46 | 0.998 | 0.846 |
| *Norops auratus* (Daudin, 1802) | 65 | 0.998 | 0.784 |
| *Norops bombiceps* (Cope, 1875) | 17 | 1 | 0.786 |
| *Norops brasiliensis* (Vanzolini & Williams, 1970) | 36 | 0.998 | 0.787 |
| *Norops chrysolepis* (Duméril & Bibron, 1837) | 63 | 1 | 0.794 |
| *Norops fuscoauratus* (D’Orbigny, 1837 in Duméril & Bibron, 1837) | 423 | 0.997 | 0.800 |
| *Norops ortonii* (Cope, 1868) | 175 | 0.997 | 0.820 |
| *Norops planiceps* (Troschel, 1848) | 51 | 1 | 0.836 |
| *Norops scypheus* (Cope, 1864) | 44 | 0.997 | 0.847 |
| *Norops tandai* (Avila-Pires, 1995) | 66 | 0.995 | 0.850 |
| *Norops trachyderma* (Cope, 1875) | 64 | 1 | 0.862 |
| **Hoplocercidae** |  |  |  |
| *Enyalioides laticeps* (Guichenot, 1855) | 53 | 1 | 0.858 |
| *Hoplocercus spinosus* Fitzinger, 1843 | 34 | 1 | 0.772 |
| **Leiosauridae** |  |  |  |
| *Enyalius leechii* (Boulenger, 1885) | 15 | 1 | 0.723 |
| **Polychrotidae** |  |  |  |
| *Polychrus liogaster* Boulenger, 1908 | 18 | 1 | 0.787 |
| **Tropiduridae** |  |  |  |
| *Plica plica* (Linnaeus, 1758) | 225 | 0.997 | 0.744 |
| *Plica u. ochrocollaris* (Spix, 1825) | 216 | 0.995 | 0.778 |
| *Plica u. umbra* (Linnaeus, 1758) | 97 | 1 | 0.772 |
| *Stenocercus fimbriatus* Ávila-Pires, 1995 | 10 | 1 | 0.851 |
| *Stenocercus roseiventris* D’Orbigny in Duméril & Bibron, 1837 | 16 | 0.996 | 0.853 |
| *Tropidurus oreadicus* Rodrigues, 1987 | 108 | 0.997 | 0.772 |
| *Uracentron a. azureum* (Linnaeus, 1758) | 24 | 1 | 0.803 |
| *Uracentron flaviceps* (Guichenot, 1855) | 16 | 1 | 0.808 |
| *Uranoscodon superciliosus* (Linnaeus, 1758) | 250 | 0.994 | 0.816 |
| **Alopoglossidae** |  |  |  |
| *Alopoglossus atriventris* Duellman, 1973 | 40 | 0.996 | 0.782 |
| *Ptychoglossus brevifrontalis* Boulenger, 1912 | 25 | 0.997 | 0.832 |
| **Gymnophthalmidae** |  |  |  |
| *Arthrosaura kockii* (Lidth de Jeude, 1904) | 41 | 0.998 | 0.812 |
| *Arthrosaura reticulata* (O’Shaughnessy, 1881) | 123 | 0.995 | 0.810 |
| *Bachia flavescens* (Bonnaterre, 1789) | 49 | 0.992 | 0.828 |
| *Cercosaura argulus* Peters, 1863 | 61 | 0.999 | 0.832 |
| *Cercosaura bassleri* Ruibal, 1952 | 31 | 1 | 0.847 |
| *Cercosaura eigenmanni* (Griffin, 1917) | 44 | 0.996 | 0.866 |
| *Cercosaura ocellata* Wagler, 1830 | 56 | 1 | 0.780 |
| *Cercosaura oshaughnessyi* (Boulenger, 1885) | 35 | 0.996 | 0.872 |
| *Colobosaura modesta* (Reinhardt & Luetken, 1862) | 39 | 0.997 | 0.792 |
| *Loxopholis guianense* (Ruibal, 1952) | 56 | 1 | 0.901 |
| *Loxopholis percarinatum* (Müller, 1923) | 111 | 0.996 | 0.880 |
| *Loxopholis snethlageae*(Avila-Pires, 1995) | 13 | 1 | 0.906 |
| *Neusticurus bicarinatus* (Linnaeus, 1758) | 62 | 0.997 | 0.910 |
| *Neusticurus rudis* Boulenger, 1900 | 17 | 0.99 | 0.921 |
| *Potamites ecpleopus* (Cope, 1875) | 79 | 0.996 | 0.809 |
| *Tretioscincus agilis* (Ruthven, 1916) | 41 | 0.996 | 0.759 |
| *Tretioscincus oriximinensis* Ávila-Pires, 1995 | 16 | 0.992 | 0.759 |
| **Teiidae** |  |  |  |
| *Ameiva ameiva* (Linnaeus, 1758) | 493 | 0.997 | 0.792 |
| *Cnemidophorus cryptus* Cole & Dessauer, 1993 | 69 | 0.999 | 0.783 |
| *Cnemidophorus lemniscatus* (Linnaeus, 1758) | 57 | 0.998 | 0.787 |
| *Crocodilurus amazonicus* Spix, 1825 | 33 | 0.994 | 0.815 |
| *Kentropyx altamazonica* (Cope, 1876) | 99 | 0.99 | 0.779 |
| *Kentropyx calcarata* Spix, 1825 | 268 | 0.997 | 0.808 |
| *Kentropyx pelviceps* Cope, 1868 | 99 | 0.998 | 0.838 |
| *Kentropyx striata* (Daudin, 1802) | 48 | 1 | 0.856 |
| *Tupinambis teguixin*(Linnaeus, 1758) | 122 | 0.996 | 0.784 |
