# Supplementary material for: Synergistic effects of climate and landscape change on the conservation of Amazonian lizards
Source: PeerJ. 2022 Mar 29;10:e13028. doi: 10.7717/peerj.13028 (PMC8973465; doi:10.7717/peerj.13028)
Supplement: Supplemental Information 2 — Variables in bold are those that were excluded due to high correlation (>0.800), also in bold. All the other variables were used in predictive models. [file peerj-10-13028-s002.docx]

**Table S2:**

**Pearson correlation of Wordclim bioclimatic variables cut to the Amazon in 2,5 arc-min resolution.**

Variables in bold are those that were excluded due to high correlation (>0.800), also in bold. All the other variables were used in predictive models.

|  | BIO1 | **BIO10** | **BIO11** | BIO12 | **BIO13** | BIO14 | **BIO15** | **BIO16** | **BIO17** | BIO18 | BIO19 | BIO2 | BIO3 | BIO4 | **BIO5** | **BIO6** | **BIO7** | **BIO8** | **BIO9** |
| --- | --- | --- | --- | --- | --- | --- | --- | --- | --- | --- | --- | --- | --- | --- | --- | --- | --- | --- | --- |
| BIO1 | 0 | **0.994** | **0.989** | 0.511 | 0.527 | 0.221 | -0.296 | 0.535 | 0.244 | 0.027 | 0.372 | -0.602 | 0.144 | -0.389 | **0.949** | **0.947** | -0.507 | **0.987** | **0.985** |
| BIO10 | 0 | 0 | 0.968 | 0.459 | 0.489 | 0.182 | -0.248 | 0.494 | 0.204 | 0.006 | 0.339 | -0.572 | 0.071 | -0.291 | 0.961 | 0.923 | -0.453 | 0.994 | 0.968 |
| BIO11 | 0 | 0 | 0 | 0.561 | 0.567 | 0.259 | -0.338 | 0.576 | 0.284 | 0.017 | 0.425 | -0.647 | 0.240 | -0.520 | 0.915 | 0.968 | -0.580 | 0.956 | 0.994 |
| BIO12 | 0 | 0 | 0 | 0 | **0.830** | 0.707 | -0.673 | **0.857** | 0.747 | 0.474 | 0.673 | -0.603 | 0.494 | -0.581 | 0.356 | 0.621 | -0.632 | 0.441 | 0.567 |
| BIO13 | 0 | 0 | 0 | 0 | 0 | 0.301 | -0.248 | **0.986** | 0.340 | 0.194 | 0.614 | -0.473 | 0.276 | -0.501 | 0.450 | 0.582 | -0.461 | 0.456 | 0.579 |
| BIO14 | 0 | 0 | 0 | 0 | 0 | 0 | **-0.843** | 0.315 | **0.991** | 0.546 | 0.510 | -0.544 | 0.678 | -0.375 | 0.005 | 0.388 | -0.640 | 0.186 | 0.270 |
| BIO15 | 0 | 0 | 0 | 0 | 0 | 0 | 0 | -0.272 | **-0.863** | -0.504 | -0.441 | 0.577 | -0.589 | 0.442 | -0.090 | -0.446 | 0.642 | -0.253 | -0.341 |
| BIO16 | 0 | 0 | 0 | 0 | 0 | 0 | 0 | 0 | 0.355 | 0.235 | 0.616 | -0.462 | 0.278 | -0.517 | 0.459 | 0.584 | -0.454 | 0.463 | 0.583 |
| BIO17 | 0 | 0 | 0 | 0 | 0 | 0 | 0 | 0 | 0 | 0.545 | 0.545 | -0.574 | 0.677 | -0.393 | 0.026 | 0.416 | -0.664 | 0.205 | 0.297 |
| BIO18 | 0 | 0 | 0 | 0 | 0 | 0 | 0 | 0 | 0 | 0 | -0.033 | -0.031 | 0.156 | -0.023 | -0.043 | 0.013 | -0.070 | 0.061 | -0.027 |
| BIO19 | 0 | 0 | 0 | 0 | 0 | 0 | 0 | 0 | 0 | 0 | 0 | -0.624 | 0.578 | -0.477 | 0.209 | 0.549 | -0.678 | 0.285 | 0.469 |
| BIO2 | 0 | 0 | 0 | 0 | 0 | 0 | 0 | 0 | 0 | 0 | 0 | 0 | -0.528 | 0.536 | -0.350 | -0.805 | **0.944** | -0.544 | -0.678 |
| BIO3 | 0 | 0 | 0 | 0 | 0 | 0 | 0 | 0 | 0 | 0 | 0 | 0 | 0 | -0.687 | -0.112 | 0.388 | -0.771 | 0.055 | 0.249 |
| BIO4 | 0 | 0 | 0 | 0 | 0 | 0 | 0 | 0 | 0 | 0 | 0 | 0 | 0 | 0 | -0.216 | -0.559 | 0.687 | -0.263 | -0.501 |
| BIO5 | 0 | 0 | 0 | 0 | 0 | 0 | 0 | 0 | 0 | 0 | 0 | 0 | 0 | 0 | 0 | 0.810 | -0.222 | 0.956 | 0.906 |
| BIO6 | 0 | 0 | 0 | 0 | 0 | 0 | 0 | 0 | 0 | 0 | 0 | 0 | 0 | 0 | 0 | 0 | -0.751 | 0.902 | 0.977 |
| BIO7 | 0 | 0 | 0 | 0 | 0 | 0 | 0 | 0 | 0 | 0 | 0 | 0 | 0 | 0 | 0 | 0 | 0 | -0.425 | -0.606 |
| BIO8 | 0 | 0 | 0 | 0 | 0 | 0 | 0 | 0 | 0 | 0 | 0 | 0 | 0 | 0 | 0 | 0 | 0 | 0 | 0.948 |
| BIO9 | 0 | 0 | 0 | 0 | 0 | 0 | 0 | 0 | 0 | 0 | 0 | 0 | 0 | 0 | 0 | 0 | 0 | 0 | 0 |
