# Supplementary figures and images for: Synergistic effects of climate and landscape change on the conservation of Amazonian lizards
Source: PeerJ. 2022 Mar 29;10:e13028. doi: 10.7717/peerj.13028 (PMC8973465; doi:10.7717/peerj.13028)

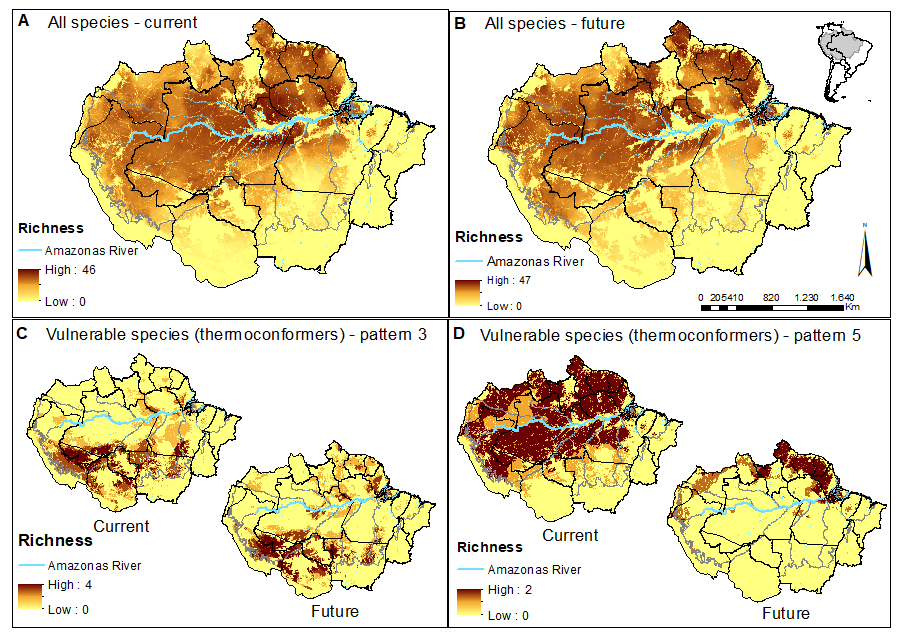

Supplement: Supplemental Information 3 — (A) Species richness based on the sum of habitat areas for all Brazilian species in the current and (B) in a future optimistic scenario, depicted by a gradient of colours, from yellow (no overlap) to brown (maximum overlap of species). The same approach only considering thermoconformers’ vulnerable species classified on (C) pattern 3 and (D) pattern 5. Blue highlight corresponds to the Amazon river. [file peerj-10-13028-s003.png]
