# Supplementary material for: Synergistic effects of climate and landscape change on the conservation of Amazonian lizards
Source: PeerJ. 2022 Mar 29;10:e13028. doi: 10.7717/peerj.13028 (PMC8973465; doi:10.7717/peerj.13028)
Supplement: Supplemental Information 4 — The graphs show the frequency (y axis) of landscapes with varying amount of habitat (%, x axis). The lines represent the analyzed scenarios: current, pessimistic future and optimistic future. [file peerj-10-13028-s004.docx]

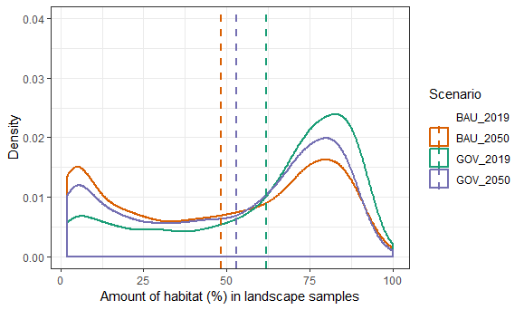


*A. atriventris*

Current

Pessimistic - Future

Optimistic - Future


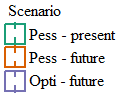

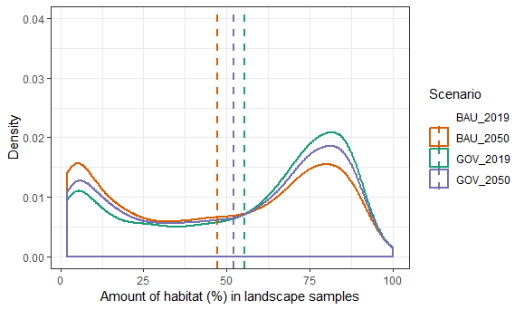


*A. ameiva*


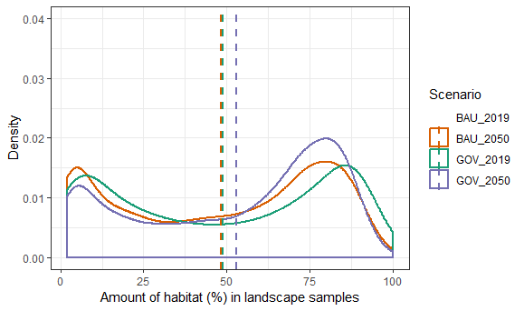


*A. kockii*


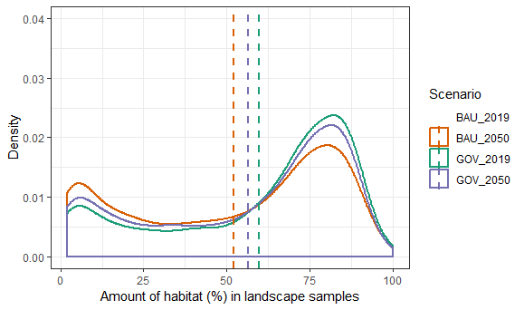


*A. reticulata*


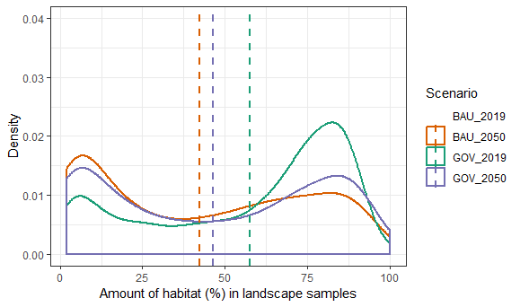


*B. flavescens*


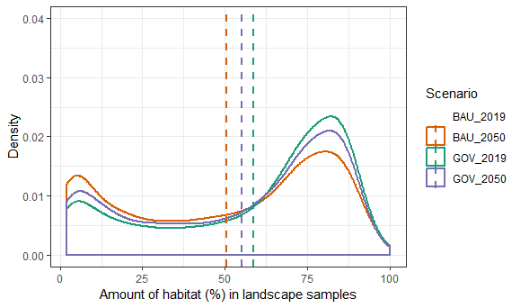


*C. argulus*


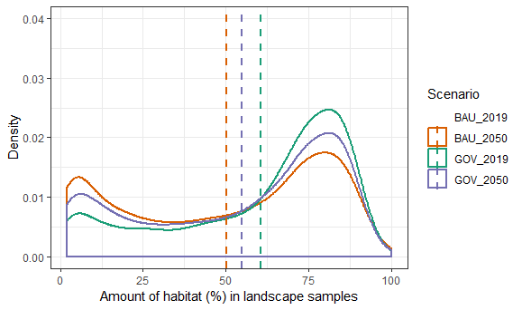


*C. bassleri*


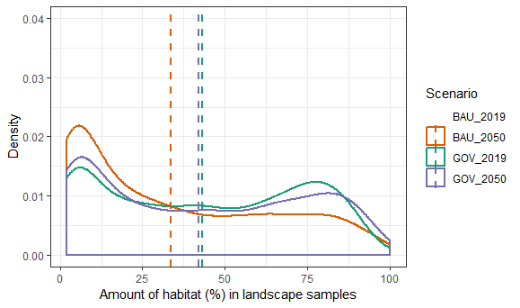


*C. eigenmanni*


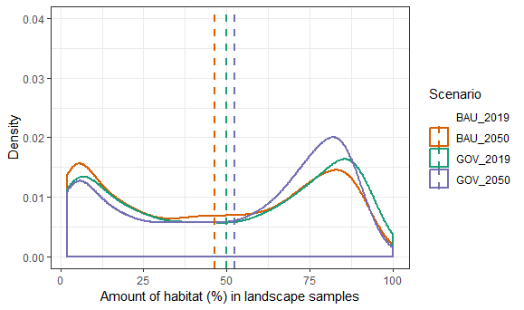


*C. ocellata*


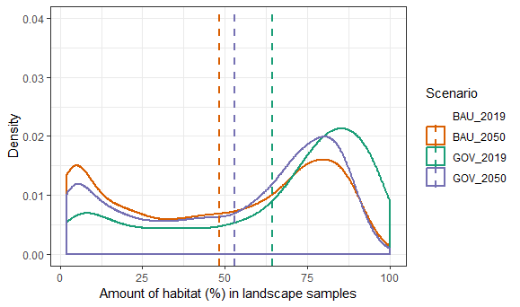


*C. oshaughnessyi*


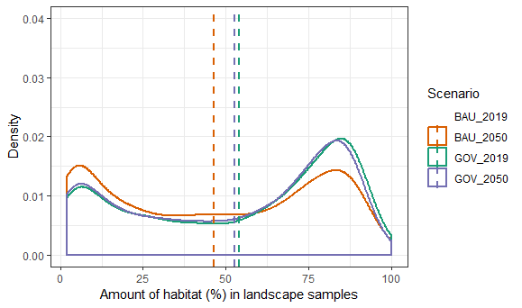


*C. amazonicus*


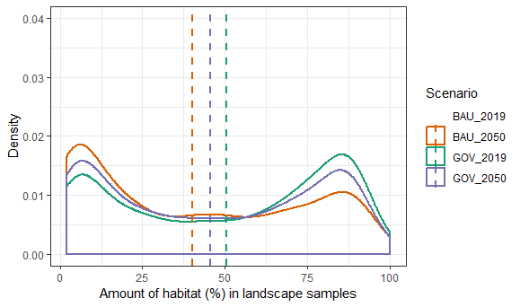


*C. cryptus*


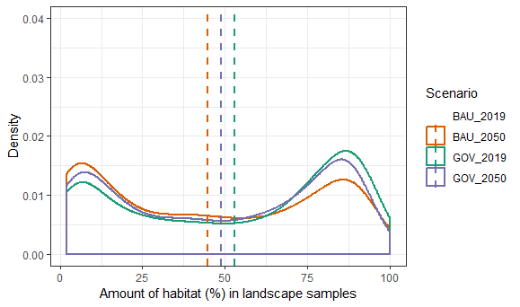


*C. lemniscatus*


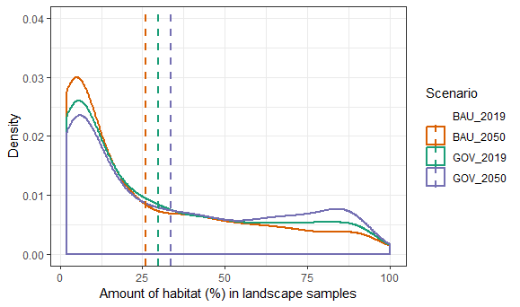


*C. modesta*


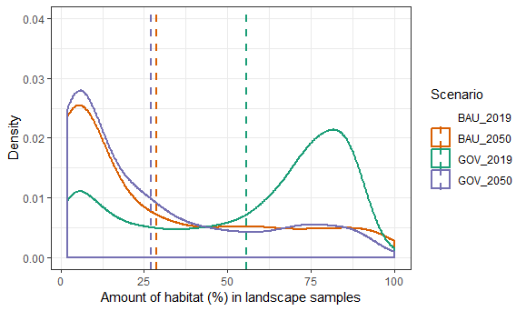


*C. nigropunctatum*


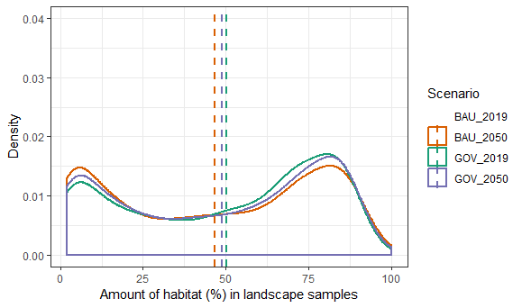


*C. amazonicus*


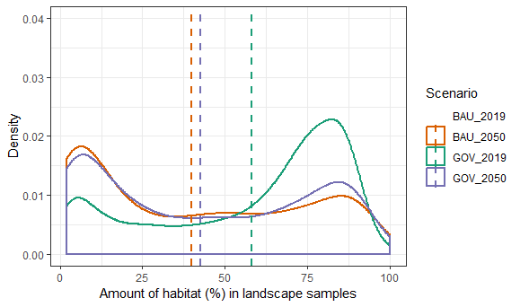


*D. punctata*


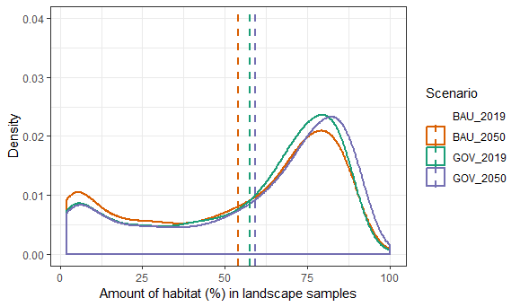


*D. transversalis*


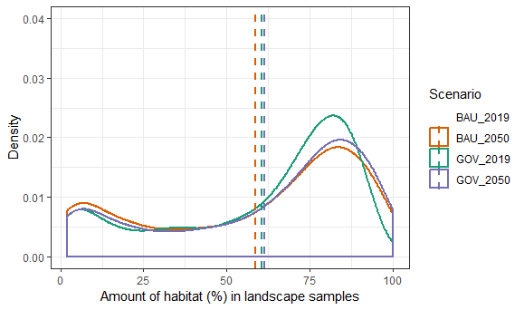


*E. laticeps*


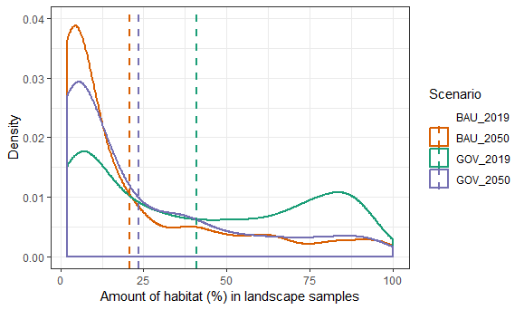


*E. leechii*


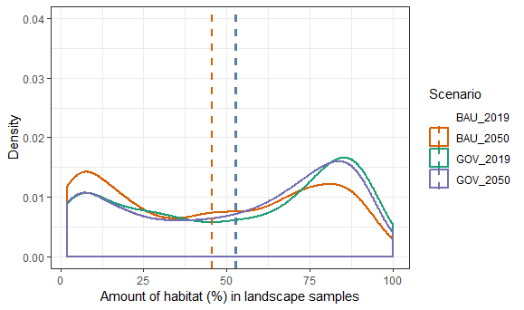


*G. annularis*


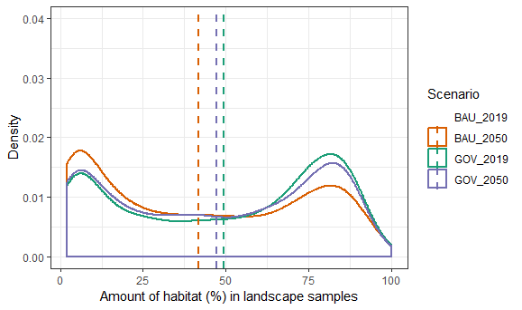


*G. hasemani*


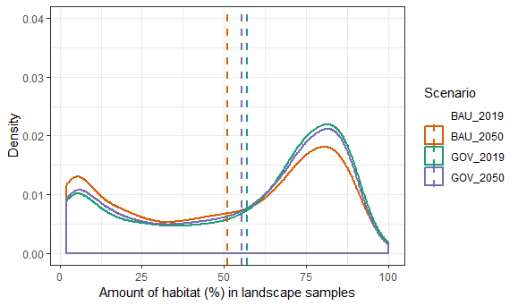


*G. humeralis*


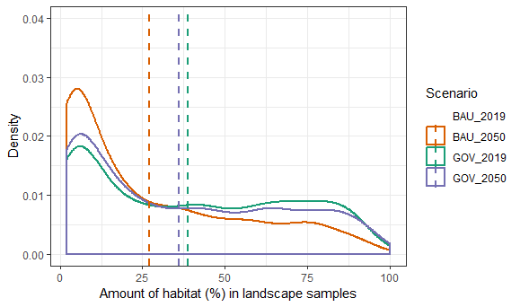


*H. spinosus*


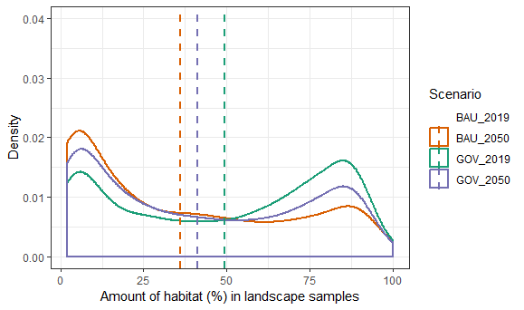


*K. calcarata*


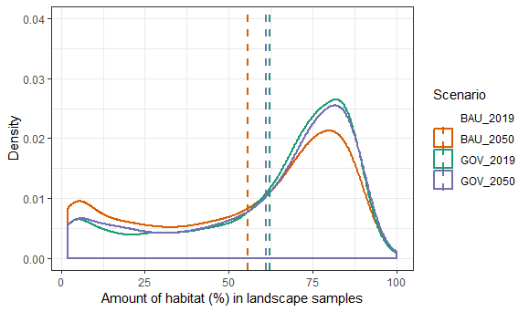


*K. pelviceps*


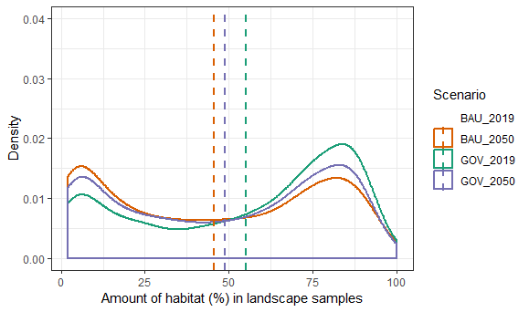


*K. striata*


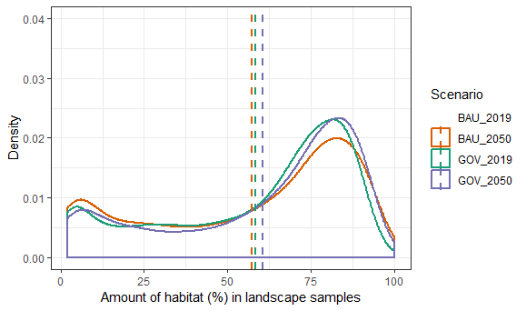


*K. altamazonica*


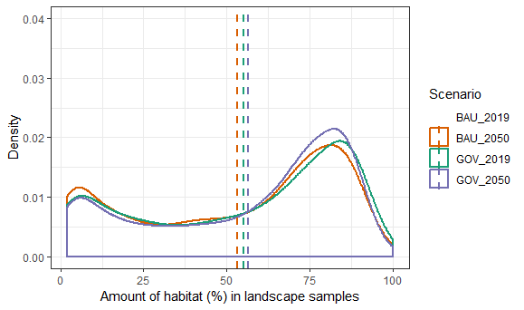


*L. heyerorum*


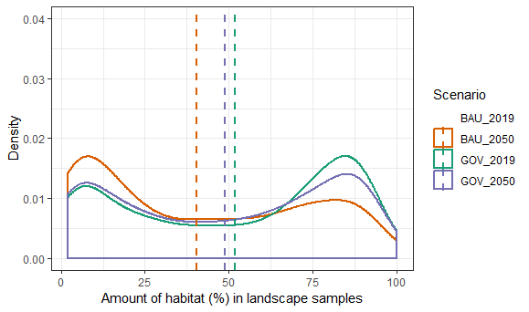


*L. guianense*


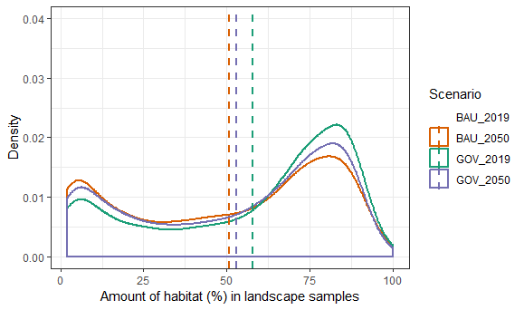


*L. percarinatum*


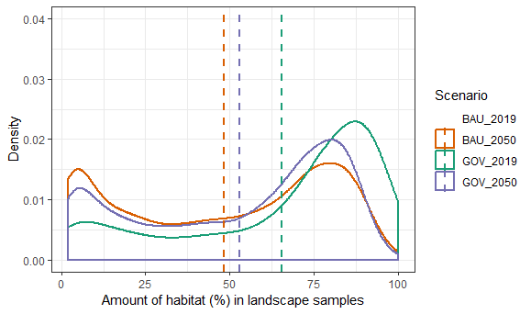


*L. snethlageae*


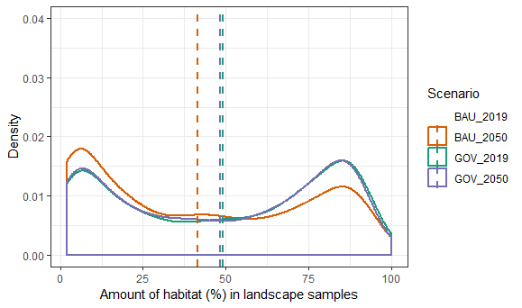


*N. bicarinatus*


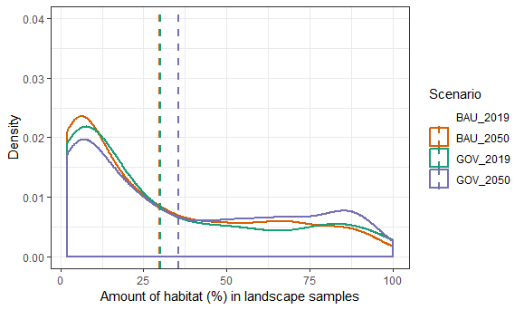


*N. rudis*


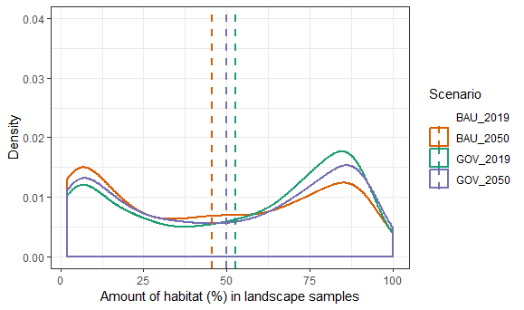


*N. auratus*


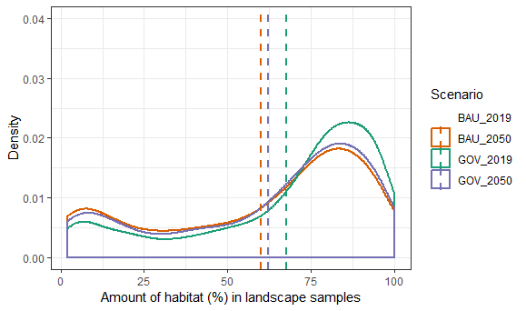


*N. bombiceps*


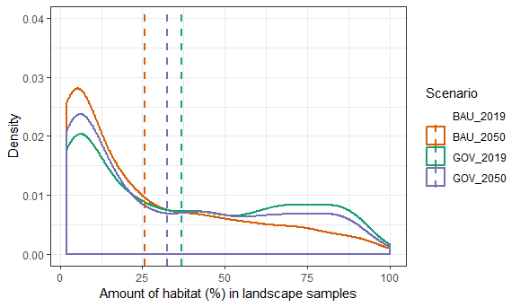


*N. brasiliensis*


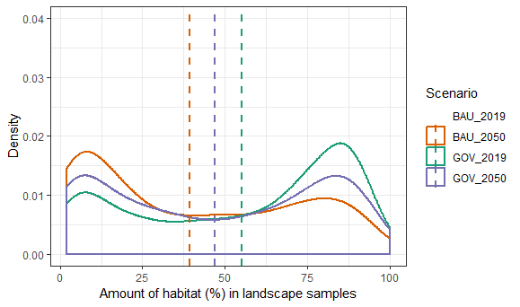


*N. chrysolepis*


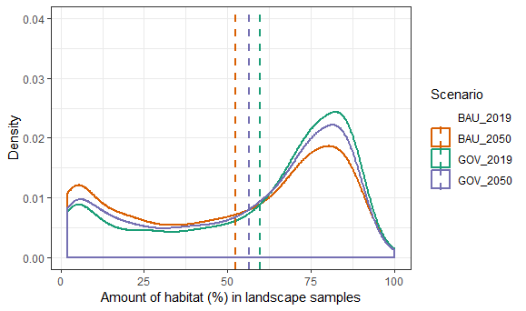


*N. fuscoauratus*


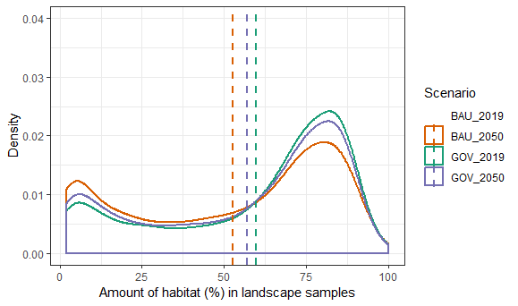


*N. ortonii*


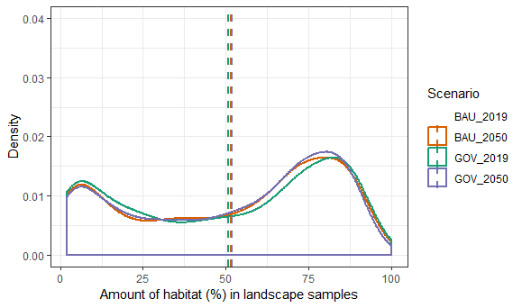


*N. planiceps*


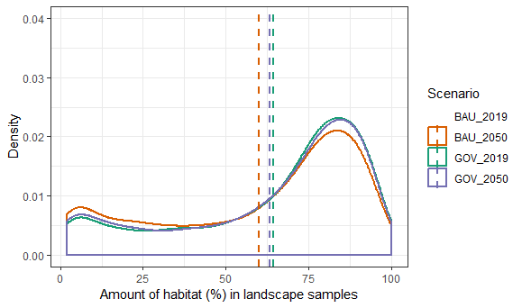


*N. scypheus*


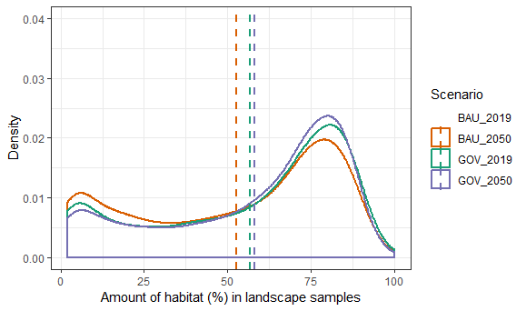


*N. tandai*


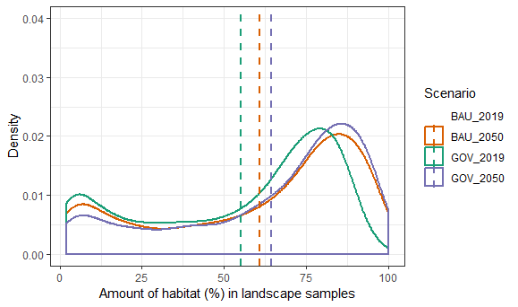


*N. trachyderma*


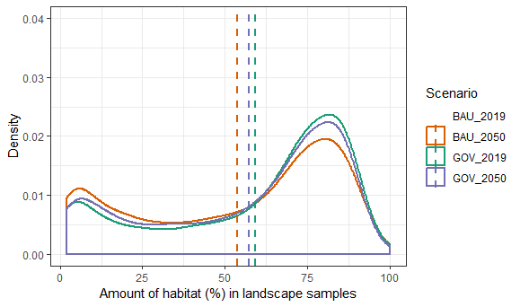


*P. plica*


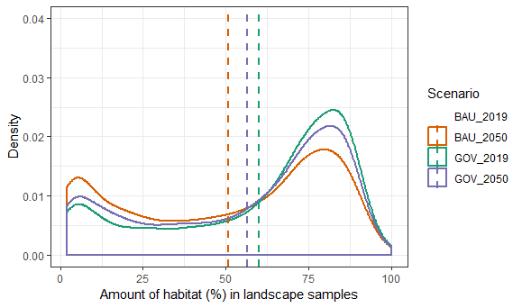


*P. u. ochrocollaris*


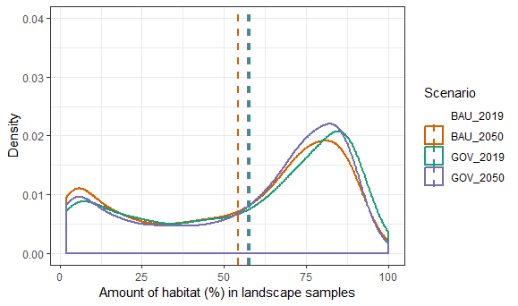


*P. u. umbra*


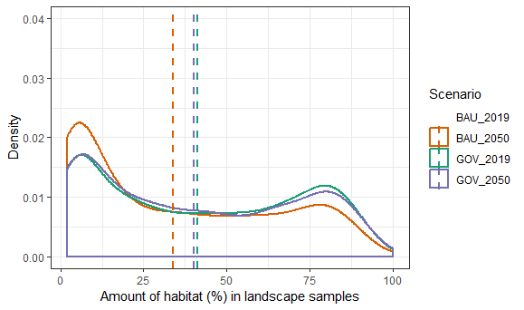


*P. liogaster*


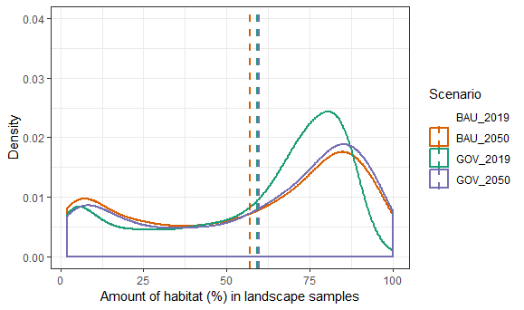


*P. ecpleopus*


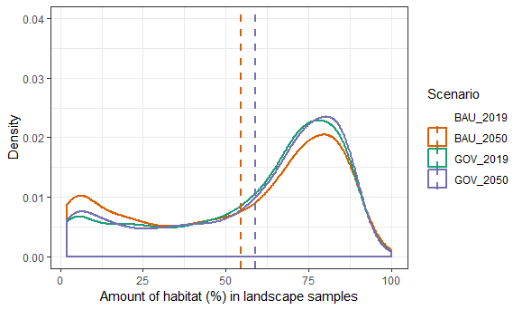


*P. guianensis*


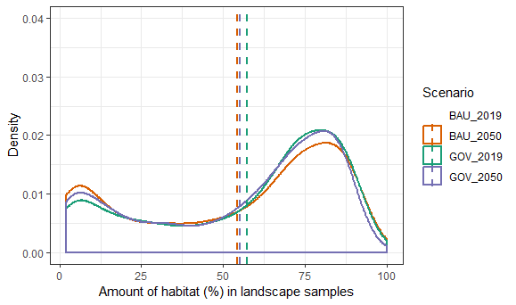


*P. brevifrontalis*


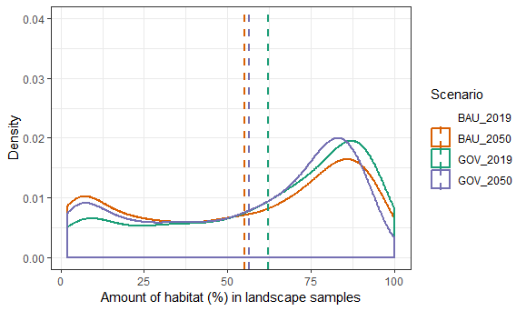


*S. fimbriatus*


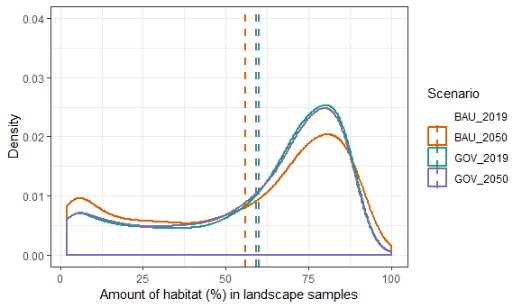


*T. solimoensis*


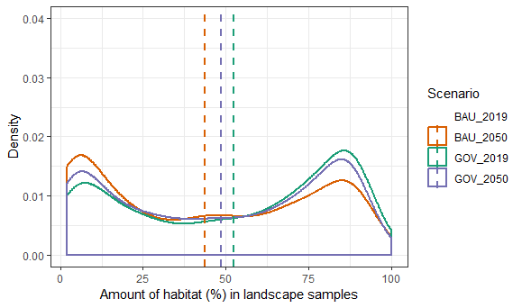


*T. agilis*


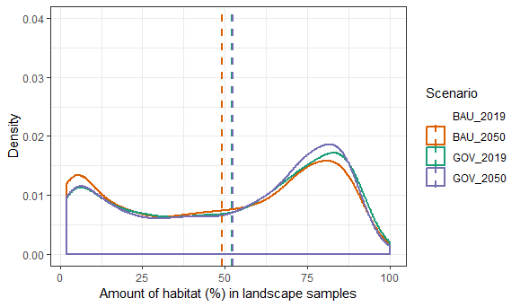


*T. oriximinensis*


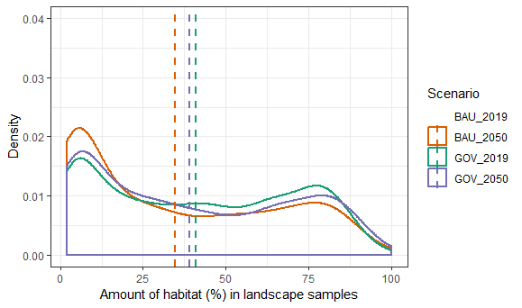


*S. roseiventris*

Current

Pessimistic - Future

Optimistic - Future


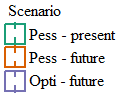

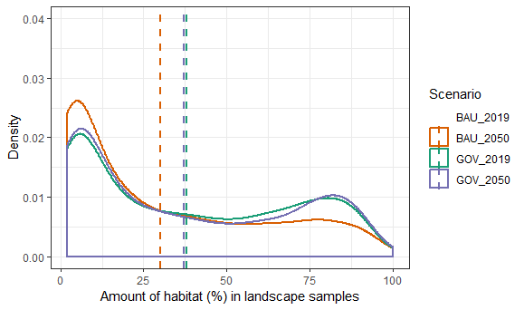


*T. oreadicus*


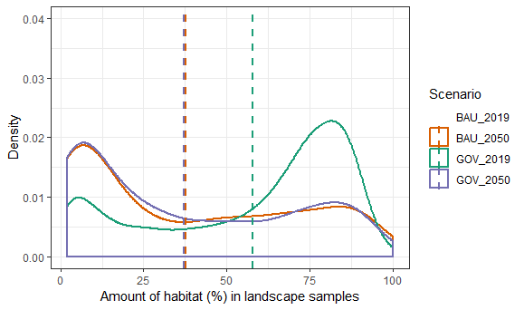


*T. teguixin*


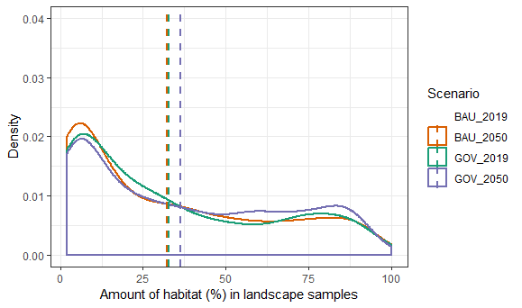


*U. a. azureum*


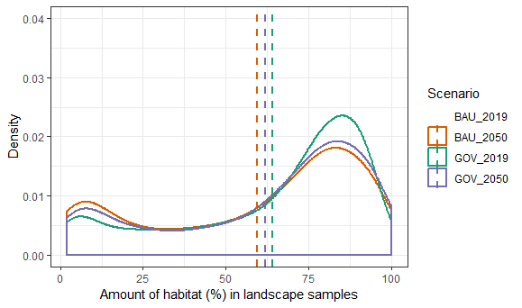


*U. flaviceps*


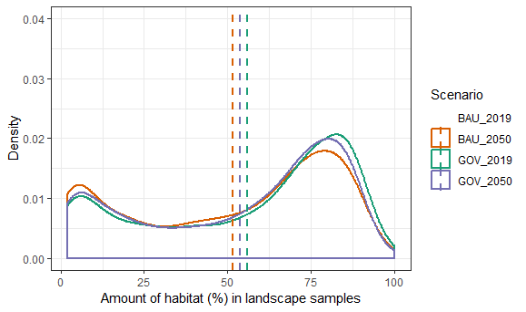


*U. superciliosus*


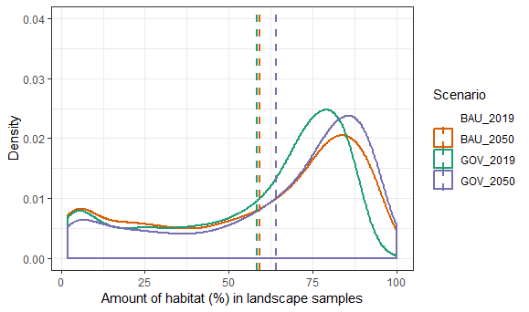


*V. altamazonica*


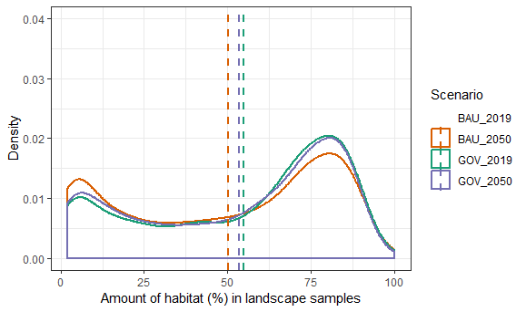


*V. bistriata*

Current

Pessimistic - Future

Optimistic - Future


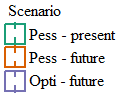


**Figure S2. Density plots for each species.**

The graphs shows the landscape density (y axis) by amount of habitat (%, x axis). The lines represent the analyzed scenarios: current, pessimistic future and optimistic future.
