# Supplementary material for: Synergistic effects of climate and landscape change on the conservation of Amazonian lizards
Source: PeerJ. 2022 Mar 29;10:e13028. doi: 10.7717/peerj.13028 (PMC8973465; doi:10.7717/peerj.13028)
Supplement: Supplemental Information 5 — Lizards species (Taxa) and total number of cells of predicted habitat areas (N° Total) in current, optimistic future and pessimistic future scenarios; number of cells in these areas when considering only landscapes >30% of the habitat area (N°>=30%) and number of cells in Protected areas (P) and their % (% P). [file peerj-10-13028-s005.docx]

**Table S3:**

**Cell number of total predicted habitat areas and stable predicted habitat areas for Amazonian lizards.**

Lizards species (Taxa) and total number of cells of predicted habitat areas (N° Total) in current, optimistic future and pessimistic future scenarios; number of cells in these areas when considering only landscapes > 30% of the habitat area (N°>=30%) and number of cells in Protected areas (P) and their % (% P).

|  | **Current** | | | | **Future Pessimistic** | | | | **Future Optimistic** | | | |
| --- | --- | --- | --- | --- | --- | --- | --- | --- | --- | --- | --- | --- |
| **Taxa** | **N° Total** | **N°>=30%** | **P** | **% P** | **N° Total** | **N° >=30%** | **P** | **% P** | **N° Total** | **N° >=30%** | **P** | **% P** |
| **Phyllodactylidae** |  |  |  |  |  |  |  |  |  |  |  |  |
| *T. solimoensis* | 124264 | 122189 | 56633 | 46 | 101400 | 96468 | 47081 | 49 | 116856 | 112735 | 53815 | 48 |
| **Sphaerodactylidae** |  |  |  |  |  |  |  |  |  |  |  |  |
| *C. amazonicus* | 96667 | 93771 | 54140 | 58 | 72353 | 66550 | 39854 | 60 | 102790 | 96692 | 58035 | 60 |
| *G. annularis* | 31928 | 30922 | 16567 | 54 | 30414 | 27663 | 13674 | 49 | 41800 | 39306 | 20690 | 53 |
| *G. hasemani* | 94374 | 90882 | 46372 | 51 | 61640 | 55370 | 29816 | 54 | 85659 | 79161 | 41196 | 52 |
| *G. humeralis* | 145048 | 141719 | 70297 | 50 | 119071 | 111758 | 58122 | 52 | 154429 | 146926 | 78970 | 54 |
| *L. heyerorum* | 84418 | 82150 | 43202 | 53 | 100220 | 94873 | 48024 | 51 | 125081 | 119453 | 61587 | 52 |
| *P. guianensis* | 93963 | 92455 | 43924 | 48 | 97425 | 92412 | 46890 | 51 | 116421 | 112307 | 57422 | 51 |
| **Mabuyidae** |  |  |  |  |  |  |  |  |  |  |  |  |
| *C. nigropunctatum* | 179374 | 174756 | 88414 | 51 | 10294 | 8242 | 4189 | 51 | 16432 | 12577 | 6217 | 49 |
| *V. altamazonica* | 116527 | 114581 | 54967 | 48 | 82946 | 79053 | 38498 | 49 | 92361 | 89103 | 42006 | 47 |
| *V.bistriata* | 101889 | 99308 | 57728 | 58 | 96744 | 90644 | 44402 | 49 | 121635 | 114954 | 47730 | 42 |
| **Dactyloidae** |  |  |  |  |  |  |  |  |  |  |  |  |
| *D. punctata* | 204864 | 200582 | 101387 | 51 | 26466 | 23610 | 9971 | 42 | 35918 | 32235 | 15557 | 48 |
| *D. transversalis* | 110531 | 108314 | 50712 | 47 | 88421 | 84084 | 40560 | 48 | 100649 | 96694 | 45776 | 47 |
| *N. auratus* | 62859 | 60772 | 30840 | 51 | 35671 | 32720 | 17172 | 52 | 51534 | 47796 | 26363 | 55 |
| *N. bombiceps* | 39162 | 38805 | 16823 | 43 | 29223 | 27923 | 11917 | 43 | 30335 | 29174 | 12199 | 42 |
| *N. brasiliensis* | 37122 | 34184 | 20983 | 61 | 12697 | 9792 | 8701 | 89 | 25965 | 22060 | 13938 | 63 |
| *N. chrysolepis* | 52251 | 50738 | 25872 | 51 | 17149 | 15009 | 8346 | 56 | 25021 | 22943 | 13521 | 59 |
| *N. fuscoauratus* | 191089 | 187712 | 94089 | 50 | 124960 | 117801 | 62068 | 53 | 158299 | 151339 | 80763 | 53 |
| *N. ortonii* | 165922 | 162642 | 81294 | 50 | 124988 | 118099 | 60825 | 52 | 159403 | 152467 | 80621 | 53 |
| *N. planiceps* | 63894 | 61605 | 31672 | 51 | 53659 | 50610 | 23594 | 47 | 63875 | 60172 | 28584 | 48 |
| *N. scypheus* | 83102 | 82117 | 35674 | 43 | 80766 | 77190 | 36234 | 47 | 87584 | 84487 | 38328 | 45 |
| *N. tandai* | 107348 | 105152 | 55231 | 53 | 90752 | 85674 | 43280 | 51 | 111254 | 106965 | 55033 | 51 |
| *N. trachyderma* | 104625 | 101983 | 50641 | 50 | 66071 | 63279 | 30063 | 48 | 70759 | 68322 | 31684 | 46 |
| **Hoplocercidae** |  |  |  |  |  |  |  |  |  |  |  |  |
| *E. laticeps* | 81976 | 80621 | 37443 | 46 | 40671 | 38675 | 16092 | 42 | 42274 | 40504 | 16543 | 41 |
| *H. spinosus* | 37416 | 35081 | 18017 | 51 | 19674 | 15709 | 10442 | 66 | 34025 | 29231 | 16282 | 56 |
| **Leiosauridae** |  |  |  |  |  |  |  |  |  |  |  |  |
| *E. leechii* | 31049 | 29092 | 20136 | 69 | 2851 | 2360 | 2212 | 94 | 5478 | 4116 | 3168 | 77 |
| **Polychrotidae** |  |  |  |  |  |  |  |  |  |  |  |  |
| *P. liogaster* | 46630 | 44274 | 19227 | 43 | 28587 | 24497 | 13924 | 57 | 39702 | 34999 | 17862 | 51 |
| **Tropiduridae** |  |  |  |  |  |  |  |  |  |  |  |  |
| *P. plica* | 165586 | 162401 | 81565 | 50 | 110117 | 104310 | 52707 | 51 | 135580 | 129832 | 66460 | 51 |
| *P. u. ochrocollaris* | 182912 | 179656 | 96566 | 54 | 127964 | 119887 | 66280 | 55 | 169860 | 162247 | 90525 | 56 |
| *P. u. umbra* | 68298 | 66692 | 34623 | 52 | 101754 | 96693 | 49294 | 51 | 127276 | 121989 | 63956 | 52 |
| *S. fimbriatus* | 33883 | 33411 | 15414 | 46 | 45532 | 42633 | 20813 | 49 | 52375 | 49575 | 23096 | 47 |
| *S. roseiventris* | 42923 | 40567 | 17567 | 43 | 23284 | 19929 | 10450 | 52 | 29505 | 25761 | 11964 | 46 |
| *T. oreadicus* | 51832 | 48146 | 28260 | 59 | 26357 | 21723 | 15428 | 71 | 49187 | 42913 | 28087 | 65 |
| *U. a. azureum* | 13109 | 11841 | 6612 | 56 | 16753 | 14094 | 8642 | 61 | 28191 | 24328 | 14464 | 59 |
| *U. flaviceps* | 88630 | 87548 | 40078 | 46 | 35562 | 33872 | 14838 | 44 | 37018 | 35444 | 15340 | 43 |
| *U. superciliosus* | 127959 | 124755 | 66575 | 53 | 88672 | 83600 | 41928 | 50 | 110542 | 104731 | 53324 | 51 |
| **Alopoglossidae** |  |  |  |  |  |  |  |  |  |  |  |  |
| *A. atriventris* | 104578 | 102970 | 47941 | 47 | 150623 | 140026 | 75129 | 54 | 200901 | 190424 | 101598 | 53 |
| *P. brevifrontalis* | 68067 | 66494 | 29946 | 45 | 71243 | 67665 | 32805 | 48 | 85036 | 81158 | 40300 | 50 |
| **Gymnophthalmidae** |  |  |  |  |  |  |  |  |  |  |  |  |
| *A. kockii* | 47582 | 45538 | 24857 | 55 | 150622 | 140059 | 75135 | 54 | 200901 | 190424 | 101598 | 53 |
| *A. reticulata* | 156956 | 154033 | 80563 | 52 | 116110 | 109379 | 57675 | 53 | 150764 | 144061 | 78297 | 54 |
| *B. flavescens* | 155742 | 152193 | 77703 | 51 | 28868 | 25980 | 10959 | 42 | 36911 | 33767 | 16292 | 48 |
| *C. argulus* | 161597 | 158236 | 81808 | 52 | 128246 | 120439 | 63559 | 53 | 166357 | 158328 | 85007 | 54 |
| *C. bassleri* | 137719 | 135408 | 62570 | 46 | 133522 | 125056 | 64361 | 51 | 168807 | 160505 | 82642 | 51 |
| *C. eigenmanni* | 42289 | 40354 | 22381 | 55 | 26114 | 21684 | 15259 | 70 | 41642 | 37236 | 21746 | 58 |
| *C. ocellata* | 70294 | 67526 | 39276 | 58 | 97922 | 89925 | 53095 | 59 | 140105 | 132296 | 77901 | 59 |
| *C. oshaughnessyi* | 50645 | 49981 | 22872 | 46 | 150622 | 140059 | 75135 | 54 | 200895 | 190428 | 101595 | 53 |
| *C. modesta* | 24271 | 21505 | 11559 | 54 | 17965 | 14108 | 11208 | 79 | 50757 | 30904 | 20086 | 65 |
| *L. guianense* | 42294 | 64782 | 22780 | 35 | 19450 | 17126 | 10814 | 63 | 30139 | 27763 | 17586 | 63 |
| *L. percarinatum* | 143118 | 139904 | 71600 | 51 | 94283 | 88585 | 44996 | 51 | 119805 | 113148 | 59078 | 52 |
| *L. snethlageae* | 29044 | 28674 | 13114 | 46 | 150622 | 140059 | 75135 | 54 | 200895 | 190428 | 101595 | 53 |
| *N. bicarinatus* | 73198 | 70170 | 40949 | 58 | 51900 | 46286 | 26526 | 57 | 80645 | 74433 | 44133 | 59 |
| *N. rudis* | 4599 | 4033 | 2216 | 55 | 10388 | 8339 | 4250 | 51 | 16095 | 13689 | 7628 | 56 |
| *P. ecpleopus* | 141386 | 138894 | 73001 | 53 | 42181 | 39647 | 18177 | 46 | 44098 | 41827 | 18893 | 45 |
| *T. agilis* | 64459 | 62135 | 34506 | 56 | 47883 | 43514 | 25195 | 58 | 68647 | 63495 | 37117 | 58 |
| *T. oriximinensis* | 92926 | 90040 | 48129 | 53 | 91847 | 85641 | 45412 | 53 | 118780 | 111885 | 60621 | 54 |
| **Teiidae** |  |  |  |  |  |  |  |  |  |  |  |  |
| *A. a. ameiva* | 179970 | 175475 | 87690 | 50 | 120030 | 111395 | 58520 | 53 | 161960 | 152536 | 81316 | 53 |
| *C. cryptus* | 75127 | 72230 | 42707 | 59 | 38418 | 34156 | 20826 | 61 | 60026 | 54793 | 33189 | 61 |
| *C. l. lemniscatus* | 49854 | 48102 | 26183 | 54 | 34036 | 31077 | 17597 | 57 | 50757 | 46983 | 27470 | 58 |
| *C. amazonicus* | 85804 | 82800 | 40945 | 49 | 68567 | 63393 | 31388 | 50 | 87571 | 81385 | 41395 | 51 |
| *K. altamazonica* | 139254 | 136755 | 68469 | 50 | 94011 | 89310 | 44401 | 50 | 108445 | 104171 | 50711 | 49 |
| *K. calcarata* | 111486 | 107360 | 58536 | 55 | 37497 | 32414 | 17998 | 56 | 60130 | 53434 | 31562 | 59 |
| *K. pelviceps* | 152594 | 150539 | 74286 | 49 | 103423 | 98648 | 47950 | 49 | 119541 | 115591 | 55264 | 48 |
| *K. striata* | 87152 | 84858 | 44594 | 53 | 49994 | 45933 | 23158 | 50 | 69089 | 64190 | 33918 | 53 |
| *T. teguixin* | 174720 | 170966 | 85861 | 50 | 14151 | 12377 | 4041 | 33 | 19447 | 16789 | 6607 | 39 |
