# Supplementary material for: Synergistic effects of climate and landscape change on the conservation of Amazonian lizards
Source: PeerJ. 2022 Mar 29;10:e13028. doi: 10.7717/peerj.13028 (PMC8973465; doi:10.7717/peerj.13028)
Supplement: Supplemental Information 6 — Calculated for each species, for the pessimistic and optimistic scenarios. Effect Sizes correspond to the “shift in standard-deviation between distributions” (σ present/σ future) following Engmann & Cousineau (2011), where effect sizes are considered “small” when shifts are around 0.25 σ, “medium” around 0.50 σ, and “large” around 0.75 σ (σ = standard deviation). [file peerj-10-13028-s006.docx]

**Table S4:**

**Test statistics, p-values and effect sizes from both scenarios resulting from the Anderson-Darling test to access the variation of future distributions of habitat in landscapes in relation to the current distribution.**

Calculated for each species, for the pessimistic and optimistic scenarios. Effect Sizes correspond to the “shift in standard-deviation between distributions” (σ present / σ future) following Engmann & Cousineau (2011), where effect sizes are considered “small” when shifts are around 0.25 σ, “medium” around 0.50 σ, and “large” around 0.75 σ (σ=standard deviation).

|  |  | **Optmistic scenario** | | | **Pessimistic scenario** | | |
| --- | --- | --- | --- | --- | --- | --- | --- |
| **Species** | **Pattern** | **AD** | **p.value** | **Effect size** | **AD** | **p.value** | **Effect size** |
| *Alopoglossus atriventris* | 1 | 47.5 | 0.001 | 0.89 σ | 155 | 0.001 | 0.87 σ |
| *Arthrosaura kockii* | 1 | 30.3 | 0.001 | 1.1 σ | 34.1 | 0.001 | 1.09 σ |
| *Arthrosaura reticulata* | 1 | 13.9 | 0.001 | 0.97 σ | 93.5 | 0.001 | 0.95 σ |
| *Cercosaura argulus* | 1 | 15.5 | 0.001 | 0.98 σ | 103 | 0.001 | 0.95 σ |
| *Cercosaura bassleri* | 1 | 30.7 | 0.001 | 0.91 σ | 116 | 0.001 | 0.88 σ |
| *Cercosaura oshaughnessyi* | 1 | 29.2 | 0.001 | 0.88 σ | 63.8 | 0.001 | 0.87 σ |
| *Dactyloa transversalis* | 1 | 0.73 | 0.205 | 1.01 σ | 18.3 | 0.001 | 0.97 σ |
| *Enyalioides laticeps* | 1 | 11.4 | 0.001 | 1 σ | 23 | 0.001 | 0.97 σ |
| *Gonatodes humeralis* | 1 | 3.89 | 0.002 | 1 σ | 56.6 | 0.001 | 0.98 σ |
| *Kentropyx altamazonica* | 1 | 3.28 | 0.002 | 1.02 σ | 24.9 | 0.001 | 0.98 σ |
| *Kentropyx pelviceps* | 1 | 8.39 | 0.001 | 0.99 σ | 72.1 | 0.001 | 0.92 σ |
| *Lepidoblepharis heyerorum* | 1 | 6.1 | 0.001 | 1.04 σ | 21.1 | 0.001 | 1.02 σ |
| *Loxopholis percarinatum* | 1 | 26.7 | 0.001 | 0.98 σ | 73.3 | 0.001 | 0.98 σ |
| *Loxopholis snethlageae* | 1 | 20.4 | 0.001 | 0.92 σ | 64.8 | 0.001 | 0.9 σ |
| *Norops bombiceps* | 1 | 10.2 | 0.001 | 0.95 σ | 17.6 | 0.001 | 0.93 σ |
| *Norops fuscoauratus* | 1 | 17.1 | 0.001 | 0.98 σ | 103 | 0.001 | 0.95 σ |
| *Norops ortonii* | 1 | 9.2 | 0.001 | 0.97 σ | 82.8 | 0.001 | 0.95 σ |
| *Norops planiceps* | 1 | 3.96 | 0.002 | 1.05 σ | 6.26 | 0.001 | 1.05 σ |
| *Norops scypheus* | 1 | 0.97 | 0.103 | 0.98 σ | 12.3 | 0.001 | 0.94 σ |
| *Norops tandai* | 1 | 3.27 | 0.001 | 1.05 σ | 31.8 | 0.001 | 0.99 σ |
| *Norops trachyderma* | 1 | 12.7 | 0.001 | 1.12 σ | 6.61 | 0.001 | 1.05 σ |
| *Plica plica* | 1 | 7.38 | 0.001 | 1 σ | 54.4 | 0.001 | 0.97 σ |
| *Plica u. ochrocollaris* | 1 | 20.8 | 0.001 | 0.97 σ | 152 | 0.001 | 0.94 σ |
| *Plica u. umbra* | 1 | 9.82 | 0.001 | 1.03 σ | 32.7 | 0.001 | 1.02 σ |
| *Potamites ecpleopus* | 1 | 65.2 | 0.001 | 1.02 σ | 86.7 | 0.001 | 0.99 σ |
| *Pseudogonatodes guianensis* | 1 | 0.95 | 0.116 | 0.98 σ | 22.5 | 0.001 | 0.92 σ |
| *Ptychoglossus brevifrontalis* | 1 | 1.58 | 0.026 | 0.98 σ | 12.1 | 0.001 | 0.97 σ |
| *Stenocercus fimbriatus* | 1 | 2.53 | 0.003 | 0.94 σ | 14.7 | 0.001 | 0.92 σ |
| *Thecadactylus solimoensis* | 1 | 2.24 | 0.008 | 1.02 σ | 19 | 0.001 | 1.01 σ |
| *Tretioscincus oriximinensis* | 1 | 1.28 | 0.049 | 1 σ | 37.6 | 0.001 | 0.94 σ |
| *Uracentron flaviceps* | 1 | 14.8 | 0.001 | 0.98 σ | 26.9 | 0.001 | 0.94 σ |
| *Uranoscodon superciliosus* | 1 | 11.5 | 0.001 | 1.01 σ | 42.8 | 0.001 | 1.01 σ |
| *Varzea altamazonica* | 1 | 1.56 | 0.03 | 1.03 σ | 17.4 | 0.001 | 0.98 σ |
| *Varzea bistriata* | 1 | 1.99 | 0.015 | 1 σ | 27.5 | 0.001 | 0.97 σ |
| *Cercosaura ocellata* | 2 | 11 | 0.001 | 1.04 σ | 31.3 | 0.001 | 1.05 σ |
| *Chatogeckko amazonicus* | 2 | 5.02 | 0.001 | 1.02 σ | 58.2 | 0.001 | 1.01 σ |
| *Gonatodes annularis* | 2 | 2.21 | 0.009 | 1.04 σ | 19.1 | 0.001 | 1.03 σ |
| *Neusticurus bicarinatus* | 2 | 1.13 | 0.083 | 1.01 σ | 37.5 | 0.001 | 1.03 σ |
| *Cercosaura eigenmanni* | 3 | 4.68 | 0.001 | 1.01 σ | 65.5 | 0.001 | 1.1 σ |
| *Colobosaura modesta* | 3 | 10.4 | 0.001 | 0.92 σ | 8.51 | 0.001 | 1.08 σ |
| *Enyalius leechii* | 3 | 57.2 | 0.001 | 1.27 σ | 58.7 | 0.001 | 1.35 σ |
| *Hoplocercus spinosus* | 3 | 6.55 | 0.001 | 1.04 σ | 59.6 | 0.001 | 1.14 σ |
| *Neusticurus rudis* | 3 | 4.96 | 0.001 | 0.93 σ | 0.58 | 0.383 | 1.01 σ |
| *Norops brasiliensis* | 3 | 8.07 | 0.001 | 1.04 σ | 48.2 | 0.001 | 1.22 σ |
| *Polychrus liogaster* | 3 | 0.64 | 0.248 | 1.01 σ | 25.9 | 0.001 | 1.04 σ |
| Stenocercus roseiventris | 3 | 3.96 | 0.001 | 1.01 σ | 24.5 | 0.001 | 1.01 σ |
| *Tropidurus oreadicus* | 3 | 0.91 | 0.117 | 0.99 σ | 41.5 | 0.001 | 1.09 σ |
| *Uracentron a. azureum* | 3 | 4.02 | 0.001 | 0.97 σ | 1.27 | 0.058 | 1.01 σ |
| *Ameiva a. ameiva* | 4 | 15.6 | 0.001 | 0.98 σ | 104 | 0.001 | 0.97 σ |
| *Cnemidophorus cryptus* | 4 | 13.8 | 0.001 | 1.01 σ | 58 | 0.001 | 1.03 σ |
| *Cnemidophorus l. lemniscatus* | 4 | 4.3 | 0.001 | 1.01 σ | 22.6 | 0.001 | 1.01 σ |
| *Crocodilurus amazonicus* | 4 | 2.3 | 0.002 | 0.98 σ | 12.2 | 0.001 | 0.97 σ |
| *Gonatodes hasemani* | 4 | 4.04 | 0.002 | 1.01 σ | 49.1 | 0.001 | 1.02 σ |
| *Kentropyx striata* | 4 | 24.2 | 0.001 | 0.98 σ | 59.2 | 0.001 | 0.98 σ |
| *Loxopholis guianense* | 4 | 5.99 | 0.001 | 1.03 σ | 37.7 | 0.001 | 1.05 σ |
| *Norops auratus* | 4 | 4.82 | 0.001 | 1 σ | 22.9 | 0.001 | 1 σ |
| *Norops chrysolepis* | 4 | 23.4 | 0.001 | 0.99 σ | 66.7 | 0.001 | 1.03 σ |
| *Tretioscincus agilis* | 4 | 9.53 | 0.001 | 1 σ | 45.5 | 0.001 | 1.01 σ |
| *Bachia flavescens* | 5 | 69.8 | 0.001 | 0.91 σ | 117 | 0.001 | 0.94 σ |
| *Copeoglossum nigropunctatum* | 5 | 341 | 0.001 | 1.1 σ | 250 | 0.001 | 1.08 σ |
| *Dactyloa punctata* | 5 | 130 | 0.001 | 0.89 σ | 165 | 0.001 | 0.91 σ |
| *Kentropyx calcarata* | 5 | 44.6 | 0.001 | 1 σ | 111 | 0.001 | 1.04 σ |
| *Tupinambis teguixin* | 5 | 158 | 0.001 | 0.94 σ | 128 | 0.001 | 0.94 σ |
